# *Toxoplasma gondii* Type I, predominant genotype isolated from sheep in South of Iran

**DOI:** 10.14202/vetworld.2017.386-392

**Published:** 2017-04-07

**Authors:** Belal Armand, Kavous Solhjoo, Manoochehr Shabani Kordshooli, Mohammad Hasan Davami, Morteza Pourahmad, Vahideh Orfaee

**Affiliations:** 1 Department of Parasitology and Mycology, Jahrom University of Medical Sciences, Jahrom, Iran; 2 Department of Parasitic Disease, Zoonoses Research Center, Jahrom University of Medical Sciences, Jahrom, Iran; 3 Infectious Diseases and Tropical Medicine Research Center, Isfahan University of Medical Sciences, Isfahan, Iran; 4 Department of Biology, Basic Sciences Faculty, Islamic Azad University, Jahrom Branch, Jahrom, Iran

**Keywords:** genotyping, GRA6 gene, Iran, polymerase chain reaction-restriction fragment length polymorphism, SAG2 gene, sheep, *Toxoplasma gondii*

## Abstract

**Aim:**

This study was performed to determine the genetic diversity of *Toxoplasma gondii* in sheep using nested-polymerase chain reaction-restriction fragment length polymorphism (PCR-RFLP) in Southern Iran.

**Materials and Methods:**

The tissue samples of diaphragm and heart from 125 sheep were collected from the main slaughterhouses of Jahrom district in South of Fars province, Iran, between Aprils and June 2013. The DNA were extracted and analyzed by nested-PCR using specific primers for SAG2 and GRA6 loci. RFLP was used to classify strains into one of the three major lineages of *T. gondii*.

**Results:**

*T. gondii* Type I was predominant in this area. The data obtained from both loci demonstrated that the frequency of each genotype was 72% Type I, 2.4% Type III, 7.2% mixed Type I and II, 16.8% mixed Type I and III, 0.8% mixed Type II and III, and 0.8% mixed Type I, II and III.

**Conclusions:**

Although the previously published data indicated that Type II is the predominant *T. gondii* genotype in sheep in the other parts of the world, this study showed that genotype I is the dominant genotype of *T. gondii* in the southern Iran; however, other genotypes were detected. High diversity of *T. gondii* genotypes including mix genotypes in lambs is of importance for the public health. These studies depict a new mapping of *T. gondii* genotypes pattern which could be very helpful in toxoplasmosis control and prevention.

## Introduction

*Toxoplasma gondii* is an obligate intracellular parasite which is capable of infecting warm-blood animal including human. Livestock is known as the important source of infection for human [1]. Human could be infected by ingestion of undercooked meat containing bradyzoite form in tissue cyst or water contaminated with oocyst shed by infected cats [2]. However, it is well indicated that the complications and severity of toxoplasmosis depend on the immunological condition of individuals; recent investigations have revealed that the genetic of parasite plays an important role [3].

Most of *T. gondii* isolates have been categorized in three genotypes (I, II, III) [4,5]. This diversity has been observed in Europe, North America, and Africa, but there have been some other isolates from human and animals with more diversity in South America which has been named as atypical or exotic isolate [6,7]. Little is known about the genetic diversity of *T. gondii* among different hosts and in various geographical locations in Asia [8]. Although the difference in the genetic level among the three types is lower than 1%, the severity of them in mice is considerably different in the way that Type I has been proved to be highly malignant in murine infections and shows a 100% lethal dose (LD_100_) of just one parasite, whereas Type II and III genotypes are comparatively less virulent (LD_100_ >10^3^ parasites) [9].

However, there is no evidence that the difference in virulence observed in mice correlates with respective infections in humans. Type II has been predominantly detected in congenital toxoplasmosis [10-12]. In addition, most of the toxoplasmosis cases in immunosuppressed individuals (75%) have related to Type II [13]. Type I *T. gondii* has been widely observed in severe congenital toxoplasmosis in immunocompetent cases and also in ocular toxoplasmosis [10]. Moreover, Type III has been isolated in ocular infection [5]. Today, genotyping has a key role in biological study of genetic population and also in epidemiological surveys of *T. gondii*, as the characterization of parasite variants leads to identify the various aspects of toxoplasmosis such as pathogenicity level [3]. For this purpose, multilocus sequence typing (MLST) analysis of microsatellites by polymerase chain reaction-restriction fragment length polymorphism (PCR-RFLP) is one of the widespread methods. Investigations in America have indicated that most of *T. gondii* isolated from human belonged to Type II. Likewise, it was revealed that the majority of isolates from farm animals like sheep belonged to Type II. As a result, these animals were known as important sources of infection. Investigations in other parts of the world show similar results, for instance among 46 *T. gondii* isolated from sheep in France, 45 (98%) were genotype II and only one case belonged to Type III [14]. In addition, two studies in UK and Switzerland all the isolated *T. gondii* were classified as genotype II based on PCR-RFLP method results [15].

The previous studies indicated that SAG2 gene is an appropriate marker for classification of three genotypes because of the highly distinguishing polymorphism region in 3’ and 5’ terminals of this gene [16,17]. However, using a single genetic locus cannot help to classify the mixed isolates [18,19]. To discriminate closely related isolates and achieve high resolution using other markers seems crucial. The GRA6 gene has been widely used as a marker because it can clearly differentiate between the three *T. gondii* genotypes, as well as between some atypical genotypes [20,21].

There is not enough information about genetic diversity of *T. gondii* in Iran, especially in Fars province, in spite of the studies in the north and central regions of the country. In another way, sheep breeding is significantly common in this area, and since the contaminated lamb is one of the sources of human infection, this study was performed to determine the genetic diversity of *T. gondii* using nested-PCR-RFLP of SAG2 and GRA6 gene in sheep in South of Fars province.

## Materials and Methods

### Ethical approval

The experiment on animals including all procedures of this study was approved by the local Ethical Committee in Jahrom University of Medical Sciences.

### Study area and sheep samples

**Figure-1:**
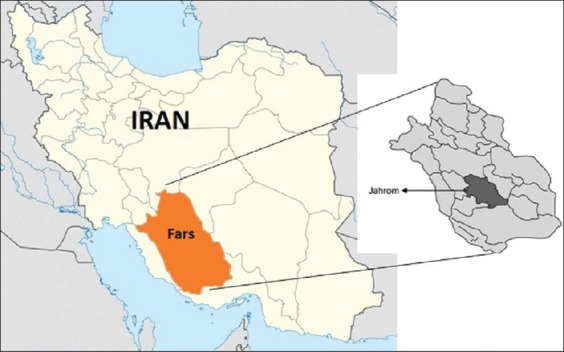
Geographical situation of the area of study, Jahrom, Fars, Iran.

Geographically, Jahrom district is located between 28.19° and 29.10° latitude north and 52.45° and 54.4° longitude east. Jahrom is situated in a zone with 1050 m height from sea level, with the vast citrus gardens, where the mean monthly temperature is 21°C. However, during the warmest period (June-August), the mean average temperature goes up to 40°C; during the cooler months (December-February), the temperature drops to below 0°C. The region has a relatively poor rainfall patterns and receives around 250 mm of rainfall annually (Figure-1).

In this study, 370 sheep blood samples were collected from major slaughterhouses of Jahrom district, between April and June 2013. The samples were analyzed by enzyme-linked immunosorbent assay to determine the seroprevalence of toxoplasmosis. The DNA was extracted from tissue samples of heart and diaphragm using phenol-chloroform DNA extraction technique, and the DNA was stored at −20°C until used. B1 gene nested-PCR detection was done to survey the tissue samples. The number of 125 confirmed *T. gondii* samples, by both serological and molecular investigation, were used in the study for genotyping.

### Nested-PCR-RFLP genotyping

Characterization of samples was carried out by nested-PCR-RFLP using the SAG2 and GRA6 markers and Bionner Company PCR-premix which has been clearly developed before.

In brief, analysis of SAG2 locus was performed by nested-PCR as described by Howe *et al*. [9]. A nested-PCR approach was used for analyzing the SAG2 locus that separately amplified the 5´ and 3´ ends of the locus. The 5´ end of the locus was amplified by standard PCR for 40 cycles with the specific primers (Table-1). The resulting amplification products were diluted 1/10 in water, and the second round of 40 cycles was performed with the internal primers (Table-1). The amplified fragments were digested with *Sau3AI*, and the restriction fragments were analyzed by agarose gel electrophoresis.

**Table-1:** Names and sequences of the PCR primer pairs used.

PCR reaction	Primer name and sequence	Annealing temperature (°C)	Product length (bp)	Restriction enzyme
5’SAG2 primary PCR	SAG2.F4: (5’-GACCTCGAACAGGAACAC-3’) SAG2.R4 (5’-GCATCAACAGTCTTCGTTGC-3’)	61	300	Sau3AI
5’SAG2 secondary PCR	SAG2.F: (5’-GAAATGTTTCAGGTTGCTGC-3’) SAG2R2: (5’-GCAAGAGCGAACTTGAACAC-3’)	61	241	
3’SAG2 primary PCR	SAG2.F3: (5’-TCTGTTCTCCGAAGTGACTCC-3’) SAG2.R3: (5’-TCAAAGCGTGCATTATCGC-3’)	63	350	HhaI
3’SAG2 secondary PCR	SAG2.F2: (5’-ATTCTCATGCCTCCGCTTC-3’) SAG2.R: (5’-AACGTTTCACGAAGGCACAC-3’)	63	221	
GRA6 primary PCR	GRA6.FO: (5’-GGCAAACAAAACGAAGTG-3’) GRA6.RO: (5’-CGACTACAAGACATAGAGTG-3’)	55	950	MseI
GRA6 secondary PCR	GRA6.F: (5’-GTAGCGTGCTTGTTGGCGA-3’) GRA6.R: (5-TACAAGACATAGAGTGCCCC-3)	58	750	

PCR=Polymerase chain reaction

The 3´ end of the locus was similarly analyzed with its specific primers (Table-1) for the initial phase of nested-PCR and the internal primers (Table-1) for the second round of amplification. The resulting products were digested with *HhaI* and separated by agarose gel electrophoresis.

In addition, for GRA6 locus two steps nested-PCR was done as clearly designed by researchers [22,23]. Briefly, the first step of amplification was done for 35 cycles with the specific primers (Table-1). The resulting amplification products were diluted 1/10 in water, and the second amplification was performed with the internal primers (Table-1). All of the products were digested with *MseI* restriction enzyme. Finally, the restriction fragments were analyzed by agarose gel electrophoresis.

## Results

### Nested-PCR results

**Figure-2:**
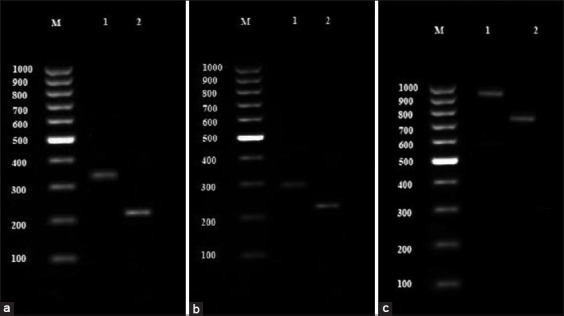
(a) Electrophoresis result of 3´ SAG2 gene nested-polymerase chain reaction (PCR) product: M – 100 bp marker, 1 – 350 bp bond from primary step product, 2 – 221 bp bond from secondary step product. (b) Electrophoresis result of 5´ SAG2 gene nested-PCR product: M – 100 bp marker, 1 – 300 bp bond from primary step product, 2 – 241 bp bond from secondary step product. (c) Electrophoresis result of 3´ GRA6 gene nested-PCR product: M – 100 bp marker, 1 – 950 bp bond from primary step product, 2 – 750 bp bond from secondary step product.

Primers were selected to separately amplify the 5´ and 3´ ends of the *T. gondii* SAG2 locus as 241 and 221 bp products, respectively, after two rounds of amplification. All the samples showed a 350 bp band in the first step and a 221 bp band after the second step of the nested-PCR; i.e., all the samples had the SAG2 gene in their 3´ terminal. Moreover, all the samples showed a 300 bp band in the initial nested-PCR step and a 241 bp band at the end of the second round; this clearly shows the presence of SAG2 gene in the 5´ terminal of the samples (Figure-2a and b).

Regarding the GRA6 gene, the electrophoresis results indicated that all the samples had a 950 bp band and 750 bp band in the first and second phase of nested-PCR, respectively (Figure-2c).

### RFLP analysis

In general, all the 125 samples, which were positive based on both SAG2 and GRA6 nested-PCR, were subjected to enzymes for RFLP step.

### RFLP analysis for SAG2 locus

**Figure-3:**
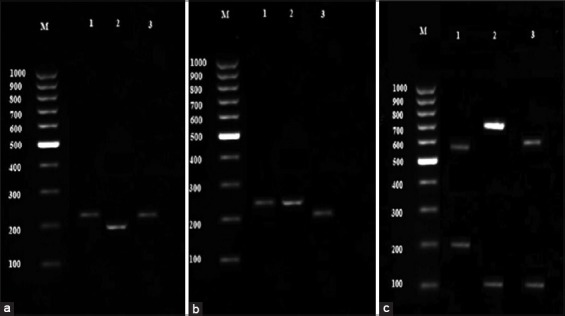
(a) Restriction fragment length polymorphism (RFLP) pattern result of 3´SAG2 gene: M – 100 bp marker, 1 - Genotype I, 2 - Genotype II, 3 - Genotype III. (b) RFLP pattern result of 5´SAG2 gene: M – 100 bp marker, 1 - Genotype I, 2 - Genotype II, 3 - Genotype III. (c) RFLP pattern result of GRA6 gene: M – 100 bp marker, 1 - Genotype I, 2 - Genotype II, 3 - Genotype III.

The amplified products of 3´ and 5´ ends of SAG2 locus were digested in *HhaI* and *Sau3AI* enzymes, respectively (Figure-3a and b). Digestion of the 5´ amplification products with *Sau3AI* distinguished allele 3 (Type III strains) from alleles 1 and 2 (Type I and II strains), and digestion of the 3´ amplification products with *HhaI* distinguished allele 2 (Type II strains) from alleles 1 and 3 (Type I and III strains). Accordingly, our results showed that four samples belonged to Type II lineage based on 3´ terminal of SAG2 gene and 16 cases were characterized as Type III based on 5´ terminals of SAG2 gene.

Combining the RFLP data obtained from 3´ to 5´ ends of SAG2 gene revealed that among 125 *T. gondii* isolated from sheep 106 samples were Type I, 4 samples Type II, 14 samples Type III and 1 sample was identified as mixed genotype of Type II and III.

### RFLP analysis for GRA6 locus

*MseI* enzyme was added to products of GRA6 nested-PCR. After incubation at 37°C for 4 h, the restriction fragments were separated by electrophoresis on agarose gel (Figure-3c). This enzyme had different cutting place in GRA6 gene in the way that it could cut the product of Type I into two fragments of 544 and 194 bp, Type II to 700 and 100 bp fragments and Type III to 600 and 100 bp fragments. The results of GRA6 RFLP indicated that the number of Type I, II and III genotypes were 105, 7 and 13 samples, respectively.

### RFLP analysis for SAG2 and GRA6

Merging the data from both loci demonstrated that genotype I (72%) was the most prevalent genotype of *T. gondii*. Furthermore, three samples (2.4%) were identified as genotype III; other samples were characterized as mixed genotype (Table-2).

**Table-2:** Overall frequency of different genotypes based on both SAG2 and GRA6 gene RFLP-genotyping.

Genotypes	Number	Frequency (%)
I	90	72
III	3	2.4
I, II	9	7.2
I, III	21	16.8
II, III	1	0.8
I, II, III	1	0.8
Total	125	100

RFLP=Restriction fragment length polymorphism

## Discussion

Today, genotyping plays an important role in biological studies as it can clarify the main pathogenic factors of an organism such as the virulence. It is clearly proved that there is a direct correlation between *T. gondii* genotype and the severity of parasite [24,25]. According to this, genotype I has been known as virulent and genotype II and III as relatively non-virulent [26]. PCR-RFLP is considered as one of the most sensitive and accurate methods for genotyping, used in many studies [27-29]. It is more sensitive than other methods in this field as it can be used in lower number of parasite in sample (5-10 parasites per sample) [30]; while other techniques such as MLST and microsatellite need at least 50 parasites per sample [31].

There are also various markers for genotyping of *T. gondii* in different researches; we chose SAG2 and GRA6 because of their several specific polymorphisms which could be very helpful for genotyping [9]. These markers have been utilized widely in *T. gondii* investigation indecently. Although each marker could precisely differentiate the genotypes, they might fail to isolate the mixed genotypes. Hence, the mixed genotypes might exist in some regions and complicate the infection symptoms [18]. Therefore, we used two markers for accurate characterization; many investigations used more markers, for the same reason, to identify *T. gondii* isolates [32,33].

Sheep is one of the major sources of meat in different countries (mainly in Iran) and is broadly consumed by people, restaurants and also in meat production companies. It has been observed that toxoplasmosis among sheep has significantly increased in the last three decades compared to other livestock like pig [34].

Seroprevalence rate of toxoplasmosis in northern and southern parts of Iran has been reported to be 55% and 29%, respectively, and a seroprevalence rate of 51.8% has been reported for all other parts of Iran [35]. Seroprevalence rate of *T. gondii* infection in Fars province has been reported to be 26.5% and 14.02% in sheep and goat, respectively [36]. Furthermore, the author’s previous study showed a 35% seroprevalence toxoplasmosis among sheep in Jahrom district [37]. This could be due to the traditional system of animal husbandry, which does not meet the standard requirements for preventing this problem. In other aspect, cats and other felids play an important role in preserving and spreading of *T. gondii* in hosts such as livestock (e.g., sheep) because they are freely wandering in the environment of animals and simply disperse the oocysts [38].

There is not enough information about genetic diversity in Iran. Our findings were similar to those who reported 66% Type I *T. gondii* in sheep in Iran (Qazvin province). They could not find any other genotypes as they used *XhoI* enzyme that can only characterize Type I only [39]. Another survey was carried out in the northern Iran in which they genotyped *T. gondii* isolated from different animals. Just four sheep isolates were genotyped which 2 cases of Type II and 2 cases of Type III were reported [40]. They properly used five microsatellites along with GRA6 marker for genotyping but because of their small samples size, their findings were not comparable with our results that from 125 samples; regardless of the differences in climate and environment of the north of Iran with Fars province. Little information is available concerning genotypes of *T. gondii* circulating in sheep worldwide. Jungersen *et al*. genotyped 11 *T. gondii* isolated from sheep (five healthy and six aborted) which all belonged to Type II [41]. Similar results were taken in the UK in the investigation of 11 isolates from aborted sheep and 2 cases of healthy sheep which all characterized as Type II *T. gondii* genotype [42]. Likewise, a survey in 2006 in France showed that all 8 *T. gondii* isolated from sheep, that genotyped based on SAG2 gene and five microsatellites, belonged to Type II [43]. Another research in France was done by Halos *et al*. in 2010; they observed that 45 cases out of 46 samples were Type II and another sample was Type III [14]. Type II was considered as predominant *T. gondii* genotype in Switzerland and China [15,44].

In summary, the previously published data indicated that Type II is the predominant strains in sheep. Interestingly, no Type I isolates of *T. gondii* has been isolated from sheep so far except one in the UK by Aspinall *et al*. [42] and Habibi [39] in Iran. To the best of our study, we analyzed 125 *T. gondii* isolates from sheep; no sampling had been done before at this level. Our findings revealed a high prevalence of Type I genotype, which has not been reported before. In addition, we found various mix genotypes which were quite different from the above-mentioned surveys. Although Aspinall *et al*. reported some mix genotypes, he suggested that the mixed types associated with food products contained mixed meat (e.g., sausages), and these results could be related to the presence of meat derived from a Type I infected animal together with that from a Type II infected animal in the same product [42].

## Conclusion

In conclusion, this study offers new and reliable perspectives about toxoplasmosis infection in lamb in Iran. High diversity of *T. gondii* genotypes including mix genotypes in lambs is of importance for the public health. Lamb, which is being widely consumed by people, could be an important source of *T. gondii* for humans. Alarmingly, it could be of a higher risk when virulent genotypes were existed in the meat products. According to our results *T. gondii* Type I is probably predominant in this area, but we could not generalize it for all parts of Iran as there was not any published data about it. Similar surveys are suggested to be undertaken for other meat-producing animals such as beef, goat, and chickens. Likewise, comparative studies should be conducted in other parts of the country and even other countries, to provide a wider insight through the animal sources of *T. gondii* for human infection. Slaughterhouses seem to be more suitable than farms for sampling since slaughtered animals are specifically processed for human food. These kinds of studies depict a new mapping of *T. gondii* genotypes pattern which can be very helpful in toxoplasmosis control and prevention.

## Authors’ Contributions

MP and KS have designed the concept and supervised the plan of work and also have prepared the manuscript. VO, BA and MSK have contributed in sample collection, administrative, technical, and material support. KS and MHD have analyzed and interpreted the data. All authors read and approved the final manuscript.
